# Bubble dynamics and atomization of acoustically levitated diesel and biodiesel droplets using femtosecond laser pulses

**DOI:** 10.1038/s41598-024-57802-8

**Published:** 2024-04-09

**Authors:** Vishal S. Jagadale, Devendra Deshmukh, Dag Hanstorp, Yogeshwar Nath Mishra

**Affiliations:** 1 Spray and Combustion Laboratory, Department of Mechanical Engineering, Indian Institute of Technology Indore, Indore, MP 453552 India; 2 Department of Physics, University of Gothenburg, 41296 Gothenburg, Sweden; 3 NASA-Jet Propulsion Laboratory, California Institute of Technology, Pasadena, CA 91109 USA; 4 Currently with Visual Computing Center, KAUST, Thuwal, Saudi Arabia

**Keywords:** Mechanical engineering, Fluid dynamics, Fossil fuels

## Abstract

This study focuses on the bubble dynamics and associated breakup of individual droplets of diesel and biodiesel under the influence of femtosecond laser pulses. The bubble dynamics were examined by suspending the droplets in the air through an acoustically levitated setup. The laser pulse energies ranged from 25 to 1050 µJ, and droplet diameters varied between 0.25 and 1.5 mm. High-speed shadowgraphy was employed to examine the influence of femtosecond laser intensity and multiple laser pulses on various spatial–temporal parameters. Four distinct sequences of regimes have been identified, depending on early and late times: bubble creation by individual laser pulses, coalescence, bubble rupture and expansion, and droplet fragmentation. At all laser intensities, early-time dynamics showed only bubble generation, while specifically at higher intensities, late-time dynamics revealed droplet breaking. The droplet breakup is further categorized into three mechanisms: steady sheet collapse, unstable sheet breakup, and catastrophic breakup, all following a well-known ligament and secondary breakup process. The study reveals that laser pulses with high repetition rates and moderate laser energy were the optimal choice for precise bubble control and cutting.

Atomization of a single droplets of diesel and biodiesel, Rapeseed Methyl Ester (RME) in a quiescent medium is crucial for understanding the fundamental physics of fluid dynamics, droplet breakup and evaporation of fuels in combustion devices. Various methods have been developed to investigate different phenomena associated with individual droplets, including fragmentation, deformation, expansion, bubble dynamics, cavitation, coalescence, and evaporation^1–6^. Laser-matter interaction, and in particular laser-induced breakdown (LIB), has emerged as a progressive technique for scrutinizing and analyzing these phenomena. The application of LIB within a single droplet has proven useful in exploring atomization processes, cleaning techniques, and biomedical applications. The nanosecond and/or femtosecond pulsed lasers or continuous laser sources such as CO_2_ lasers or fiber lasers have been used to investigate LIB in droplets^7–16^. Droplets are conventionally treated as transparent dielectrics when exposed to visible light. As the laser is focused inside the droplet, it begins to absorb the laser energy, and, when the laser intensity surpasses the breakdown threshold, plasma is generated within the droplet. Ultra-short laser pulses, particularly femtosecond pulses, play a pivotal role in controlling the dynamics of LIB.

Over the past three decades, the interaction between femtosecond pulses and droplets has yielded a multitude of applications^17–26^. When a focused femtosecond laser pulse interacts with condensed matter, it can induce optical breakdown or filamentation^19–21^. This interaction between femtosecond laser pulses and the medium encompasses various physical processes, including light absorption, non-linear ionization, plasma formation, bubble generation, thermal conduction, ablation, and electron-photon collisions. The process of generating bubbles through the focusing of multiple laser pulses comprises several steps. When high-power laser radiation at the focal point exceeds the breakdown threshold (> 10^12^ W/cm^2^)^25^, it triggers the formation of free electrons due to multiphoton ionization and tunnel ionization. Consequently, the liquid undergoes rapid excitation, ionization, and dissociation into a high temperature plasma (~ 10^4^ K) within the focal region^26^. Subsequently, recombination processes occur, and the plasma is replaced by a vaporized fluid mass, which forms micro-bubbles^11,27^. The nature of bubble formation (single or multiple, spherical or non-spherical) depends on factors such as laser energy, focusing conditions, and the properties of the liquid medium^27–29^. Laser pulse-induced bubbles are of particular importance, as they play a crucial role in cellular microsurgery^28^, removing thrombus (blood clot) from clogged arteries^30^, and clearing bile duct stones through breakup and lithotripsy^31^.

The generation of bubbles by launching the laser pulses in water^24,32–35^ and employing diverse approaches investigated in numerous studies. Potemkin and Mareev^24^ observed the evolution of multiple cavitation bubbles within a single filament stimulated by a femtosecond laser pulse in water. Laser-induced filamentation, characterized by the formation of thread-like structures due to self-focusing caused by the optical Kerr effect, played a pivotal role in this phenomenon. Expanding on this, Potemkin et al*.*^32^ explored different regimes of filamentation and the associated dynamics of shock waves and micro-bubbles induced by filaments in water. They achieved this by employing various focusing techniques, including the introduction of aberrations, modifying laser parameters such as pulse energy, and manipulating the properties of the medium, such as its linear absorption characteristics. Jukna et al*.*^33,34^ reported the significant impact of pulse duration on the shape and intensity of acoustic signals arising from the filamentation of ultrashort terawatt laser pulses in water. These signals were closely linked to the mechanism of super filamentation in water. A study by Rao et.al^35^. reported the control of the femtosecond laser induced microbubble generation in the liquid pool. The study explores the dependency of bubble size, shape and population density on laser energy, number of laser pulses and the liquid medium. Koukouvinis et al*.*^36^ examined the interaction between a laser-induced bubble and a free surface, presenting a comparison of experimental findings and Computational Fluid Dynamics (CFD) simulations utilizing the Volume of Fluid (VOF) methodology. The simulations effectively predicted bubble expansion and collapse, aligning with both qualitative and quantitative observations. In a recent study, Rosselló et al.^37^ investigated the laser-induced bubbles and jetting inside millimetric droplet. They reported on vapor bubble expansion within water droplets, acoustics secondary cavitation, and the formation of liquid jets near highly curved surfaces. Raman et al*.*^38^ studied the dynamics between a laser-generated cavitation bubble and a submillimeter-sized water droplet submerged in silicone oil, revealing three distinct interaction phases: deformation, external emulsification, and internal emulsification. Notably, during bubble collapse, the droplet elongates towards the bubble, influenced by the bubble's flow sink effect. Nevertheless, most investigations into laser-assisted bubble dynamics have primarily focused on individual bubbles, particularly at low laser energy levels (< 200 µJ). It is crucial to recognize that laser-induced cavitation bubbles^39–41^ play a pivotal role in laser-induced breakdown in liquids. Therefore, comprehending the hydrodynamics of multiple bubbles during the process is imperative. Equally significant is the examination of the interaction among micro-bubbles generated by temporally separated laser pulses. This interaction sheds light on how residual bubbles and their fragments from previous pulses interact with bubbles generated by subsequent pulses.

In this study, we employ Laser-Induced Breakdown (LIB) to investigate the fragmentation dynamics of diesel and rapeseed methyl ester (RME) fuels. While there have been numerous investigations on multi-component liquid droplets, comprehensive studies on the bubble dynamics and subsequent breakup characteristics of fuel droplets under the influence of focused multiple femtosecond laser pulses within an isolated environment are lacking in the literature. Our research addresses this gap by examining the fragmentation of acoustically levitated diesel and RME droplets of various sizes using femtosecond laser pulses with differing energies. The primary aim of this study is to elucidate the effects of laser intensity and the number of laser pulses on the process of droplet deformation, bubble dynamics, and atomization characteristics. To the best of our knowledge, our research represents the first systematic attempt to observe and analyze the entire sequence of events within a single droplet of fuel, encompassing bubble generation, growth, coalescence, rupture, droplet stretching, and eventual breakup. Given that the physio-chemical properties of biofuels differ from those of fossil fuels, their atomization processes exhibit distinctions. Understanding droplet atomization is crucial for promoting fuel flexibility, integrating renewable energy sources, and advancing technology toward sustainable and cleaner energy solutions. Experimental findings from single droplet experiments are interpreted through the application of fundamental physical principles and theoretical analyses pertaining to bubble dynamics and droplet breakup phenomena. Furthermore, our study facilitates a controlled examination of laser-induced cavitation within diesel droplets, drawing inspiration from practical diesel injectors where cavitation influences the atomization process^42,43^. In summary, this study is divided into two main parts: the first part focuses on bubble creation and dynamics, while the second part delves into the breakup of fuel droplets. Additionally, we quantify the secondary droplet size distribution of diesel and biodiesel droplets following atomization.

## Experimental methodology

**Figure 1:**
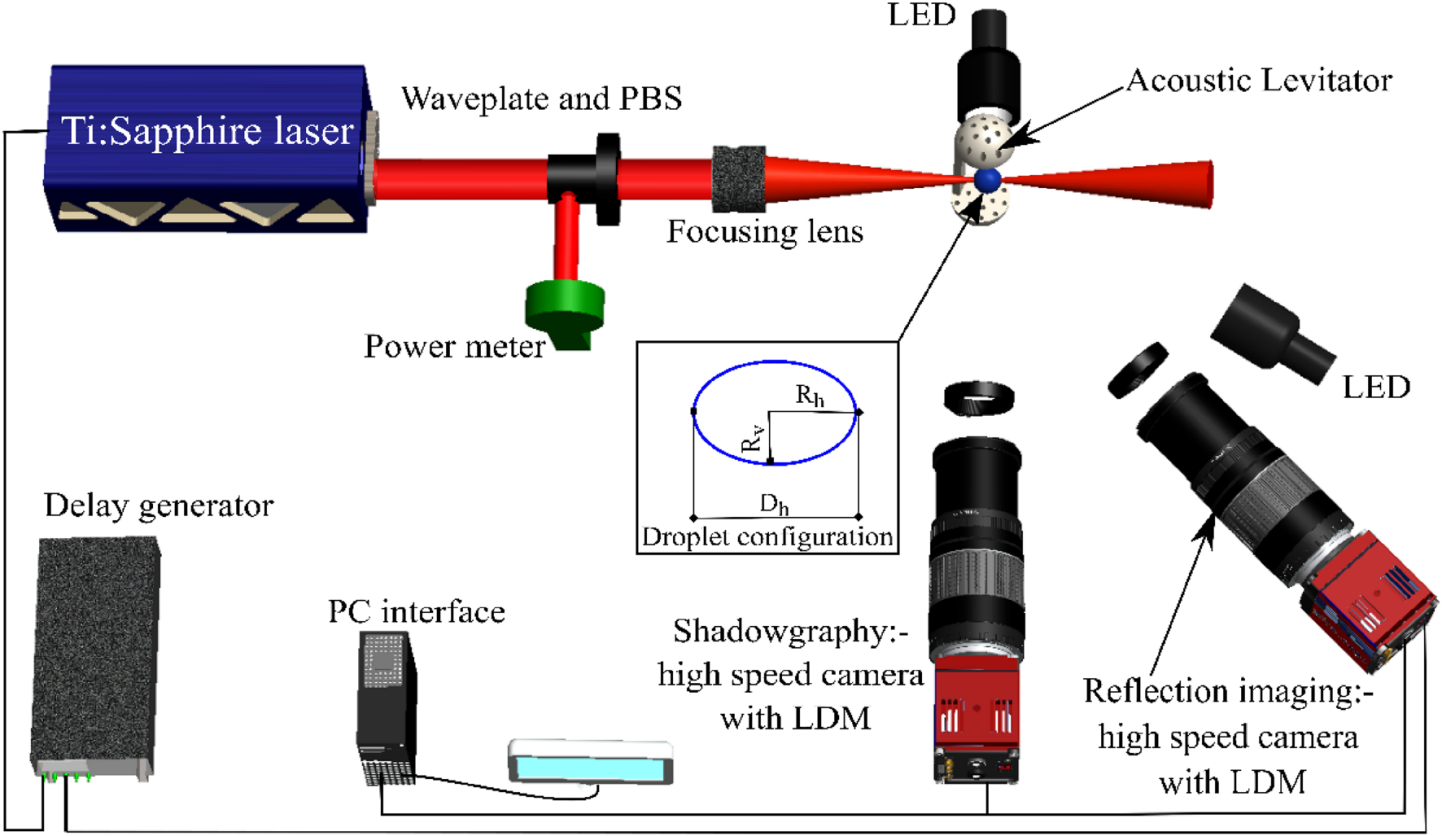
Experimental setup for femtosecond laser-induced fragmentation of acoustically levitated droplet. The inset represents the droplet configuration used for analysis.

Figure 1 provides the schematic illustration of the experimental setup used for the femtosecond laser pulse-induced breakdown in a levitated single droplet. This setup comprises three main components: a femtosecond laser source, an acoustic levitator designed for suspending a droplet within a quiescent medium, and an optical imaging system utilized for both shadowgraphy and for imaging of the reflected light. Femtosecond laser pulses are generated using an integrated Ti: Sa amplified laser system (CPA series). The laser has a beam diameter of 10 mm, a wavelength of 775 nm, a pulse duration of 150 fs, and a repetition rate of 1 kHz. The maximum energy per pulse is 1050 µJ with an average power of 1.05 W. Precise focusing of the laser beam at the center of the droplet is achieved through a near-infrared high-power doublet with a focal length of 100 mm. Laser beam polarization and energy control are managed using a combination of a quarter-wave plate (λ/4, 830 nm) and a polarizing beam splitter (PBS). Laser energy measurements are conducted near the laser exit port using an Ophir-II energy meter. The beam size near the focal point varies with laser energy and is determined by employing laser burn paper. The diameters of the laser focus spot are typically within the range 30–40 microns, i.e., considerably smaller than the size of the droplets.

The droplets are produced using a microliter syringe and needle arrangement under ambient conditions, approximately at 293 K and 50% relative humidity. A custom-built 3D-printed single-axis acoustic levitator is used to levitate the droplets. Acoustic levitation works on the principle of generating a standing acoustic wave between the emitter and reflector^44,45^. The levitator setup consists of 72 transducers of 10 mm diameter each, operating at a resonance frequency of 40 kHz^45^. The experimental investigation is conducted within a laboratory environment under standard room temperature and atmospheric pressure conditions. To ensure a precise laser beam focusing at the center of the droplet within the acoustic levitator, it is imperative to maintain the stability of the droplet^45–47^. The stability and shape of the droplet are adjusted by changing the input voltage. By lowering the voltage, the shape of the droplet is controlled near spherical, i.e., the surface tension dominates the acoustic force. Once the droplet is stable in the acoustic trap, the laser pulse is focused at the center of the droplet. Further, to ensure consistent laser energy delivery to the center of the droplet, the levitator was fixed on the 3-D stage. The equivalent onset radius of the droplet under levitation is defined as ${R}_{o}={{({R}_{h}}^{2}\times {R}_{v})}^{1/3}$, where ${R}_{h}$, ${R}_{v}$ is the horizontal and vertical radius of the droplet, respectively (droplet configuration in the inset of Fig. 1)^48,49^. Thus, ‘${D}_{o}=2 \times {R}_{o}$’ represents the onset diameter of the droplet.

The study focuses on Diesel and RME liquids. The typical properties of these liquids are compared in Table 1. RME is a mixture of saturated and unsaturated C16 to C22 fatty acids. The mixture contains methyl esters of oleic acid C18:1 (60.5%), linoleic acid C18:2 (19.8%), linolenic acid C18:3 (9.2%), and other unsaturated and saturated fatty acids in residual amounts^50–53^. The bubble dynamics and fragmentation phenomenon are recorded using two high-speed cameras (Phantom Miro LAB310 from vision research) operating at 11,000 frames per second, utilizing 512 × 512-pixel resolution and an exposure time of 18 µs. Spatial resolution is achieved by coupling both cameras with long-distance microscopes (Infinity model K2 DistaMax). Two collimated white light LEDs (Thorlabs) are used to illuminate the event. In reflection imaging, one camera captures the reflection of LED light from the droplet, while in shadowgraphy imaging, the other camera records the backlighting shadow of the droplet. The pixel resolution for shadowgraphy and reflection imaging is 6.75 µm/pixel and 11.0 µm/pixel respectively. The shadowgraph and reflection images of the droplets are subsequently subjected to post-processing and analysis using MATLAB, along with image analysis tools such as ImageJ and Image-Pro Plus. These analyses allow for the determination of bubble sizes, ligament characteristics, secondary droplet size and velocity, radial acceleration, as well as the velocity of the expanding sheet. The uncertainty in the measurement of initial droplet diameter (D_0_) and laser energy (E_l_) reported are ± 15 microns and ± 5 µJ, respectively. The experimental errors, primarily due to pixel identification, together with calibration uncertainties, accounted for no more than 1.5% of the ligament and secondary droplet size determination.

**Table 1:** Properties of the liquid samples used in the study^50–53^.

Properties	Density @293 K, kg/m^3^	Surface tension @293 K, N/m	Viscosity @293 K, mm^2^/s	Specific Heat Cp @293 K, kJ/kg K
Diesel	837	0.0258	3.04	1.75
RME	886–900	0.0332	6–9	2.47

## Results and discussion

The strength and location of Laser-Induced Breakdown (LIB) within the droplet during the interaction with the laser determine the characteristics of Opto-hydrodynamic phenomena, including fragmentation, deformation, propulsion, and breakup. These characteristics predominantly rely on the properties of the droplet, such as its optical density and breakdown threshold, as well as the distribution of laser energy within the droplet.

The distribution of laser energy inside the droplet is influenced by its optical properties, including absorption, transmission, and reflection. Consequently, the control of the opto-hydrodynamic phenomenon can be achieved by adjusting the laser's intensity or the properties of the droplet. Increasing the laser intensity can result in more pronounced effects, and vice versa, and by controlling these parameters, the opto-hydrodynamic phenomena can be tailored for different applications. The present study focuses on the interaction between femtosecond laser pulses and the dynamics of bubbles and breakup in association with diesel and RME droplets. The evolution of the droplet, from the inception of bubble formation to its eventual breakup, is categorized into three modes:

- *Bubble creation and dynamics*: This mode encompasses the formation of bubbles, their merging, and coalescence.
- *Expansion and stretching of the droplet*: Consists of the droplet's expansion and stretching.
- *Bubble rupture and sheet breakup*: This mode relates to the rupture of bubbles and the subsequent breakup of the droplet.

**Figure 2:**
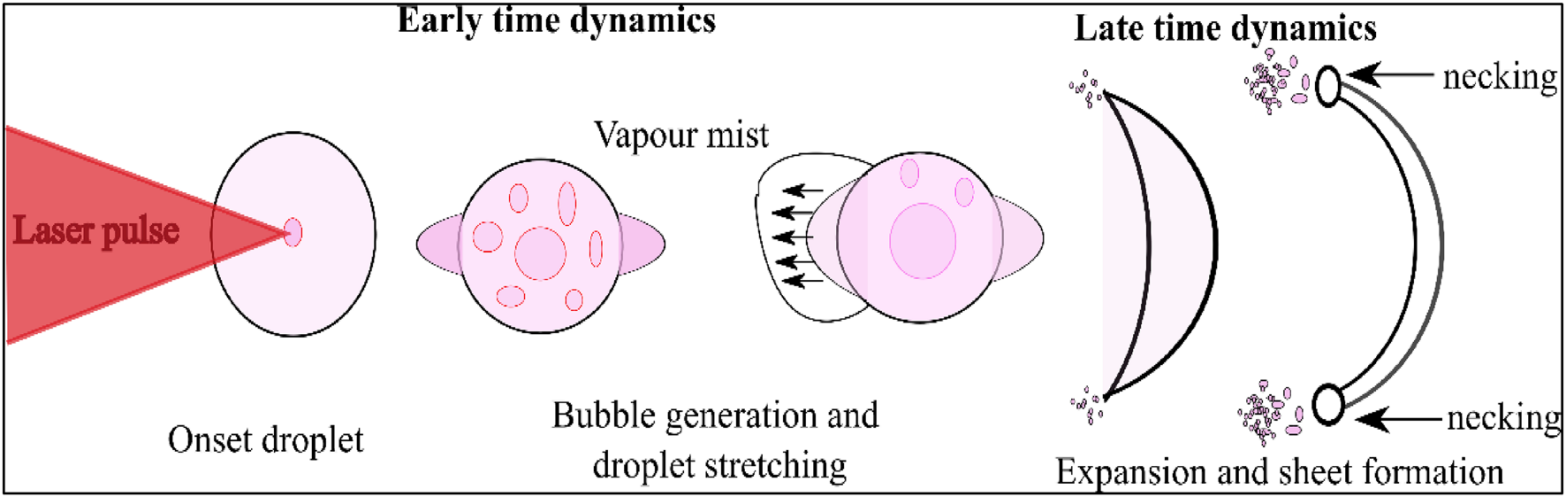
Graphical depiction of the sequential dynamics of laser-droplet interaction through multiple laser pulses.

These observed modes are explained in terms of temporal dynamics, specifically early-time and late-time dynamics, as illustrated in Fig. 2. Early-time dynamics describe the two modes: bubble and expansion dynamics, while late-time dynamics elucidate droplet rupture and sheet breakup.

### Bubble creation and dynamics

**Figure 3:**
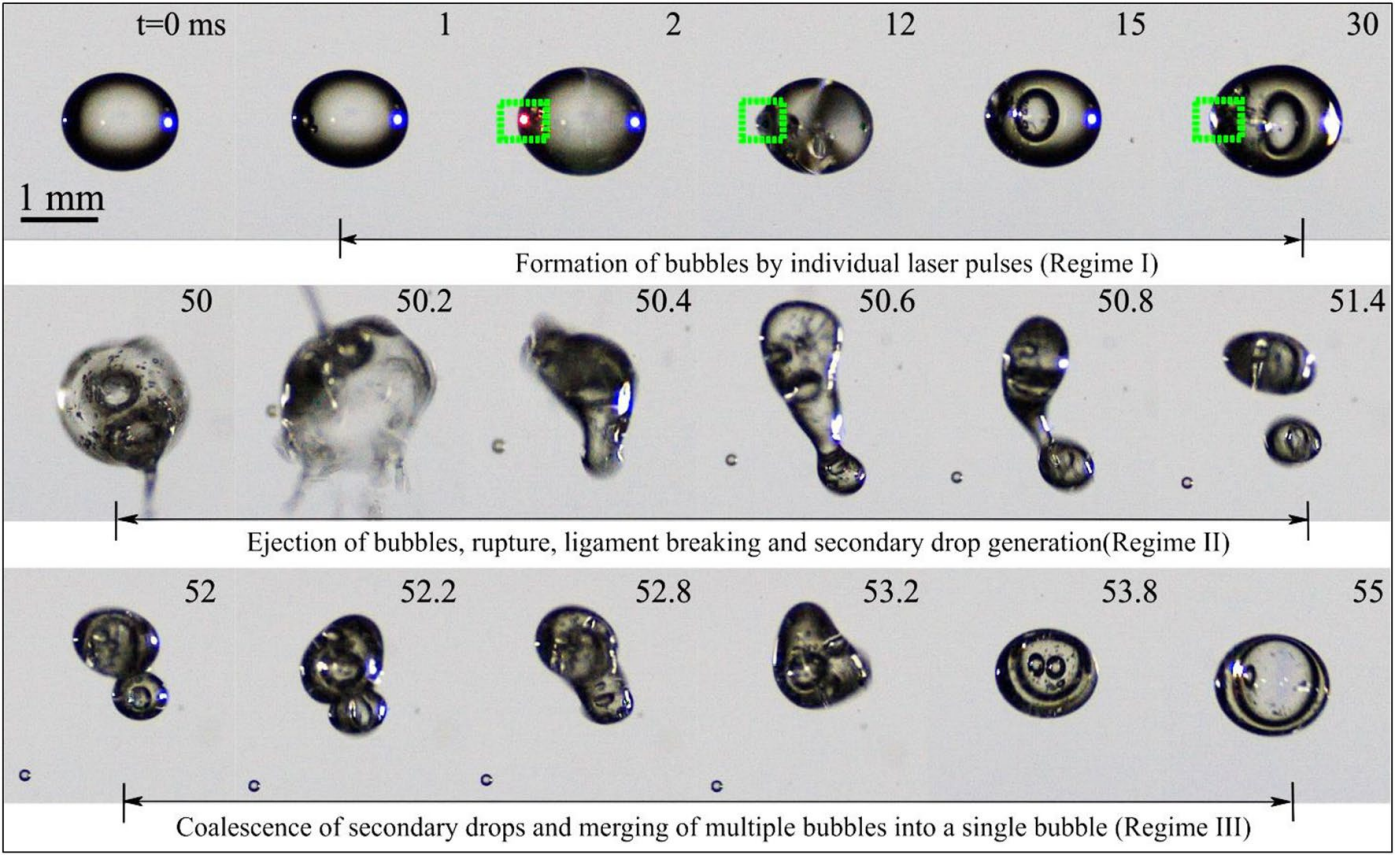
Regimes of bubble dynamics in a diesel droplet through the interaction of multiple femtosecond laser pulses at ${E}_{l}$ = 250 µJ, D_0_ = 1.4 mm. The dotted rectangles highlight the creation of bubbles by individual laser pulses.

This section delves into the dynamics of bubbles formed in diesel and RME droplets when subjected to femtosecond laser pulses. Figure 3 provides a visual representation of reflection images captured during the evolution of a diesel droplet as it interacts with a train of femtosecond laser pulses. The laser beam is focused inside the droplet, leaving the droplet's surface unaffected. Notably, the size and breakup of the resulting bubble can be controlled by adjusting the laser pulse energy and the number of pulses. At lower laser energy levels, the observed dynamics encompass the stages of bubble formation, coalescence, and rupture. The breakups strongly depend on the laser pulse energy and initial droplet size. This dependence arises from the amount of energy required to generate a sufficiently large bubble that can break the droplet. The process of bubble dynamics can be categorized into three distinct regimes:

1. *Formation of bubbles*: Regime I involves the creation of bubbles. Each laser pulse induces the formation of a single bubble within the droplet. The oscillations due to the laser pressure pulse and hydrodynamic processes, including internal recirculation, are observed during the frame interval. With multiple bubbles inside the droplet, as bubbles approach neighboring bubble, the secondary Bjerknes force causes their coalescence resulting in a single larger bubble.
2. *Rupture of bubble, ligament stretching, and droplet breaking*: In regime II, the bubble ruptures, followed by the stretching of ligaments and the subsequent breakup of the droplet. Subsequent laser pulses lead to the breakage of the merged/coalesced bubble, causing the rupture of the droplet surface, evident in Fig. 3 as jetting or splashing of bubbles from the droplet wall.
3. *Coalescence of bubbles and secondary droplets*: Regime III is characterized by the coalescence of bubbles and the formation of secondary droplets. Upon droplet surface rupture, secondary droplets are produced, as depicted in Regime II in Fig. 3. The secondary droplets formed during the breakup process may coalesce in the presence of the acoustic field used for droplet levitation.

Regime I correspond to the initial creation of small bubbles through the action of femtosecond laser pulses. Initially, multiple small bubbles emerge because of laser energy absorption. When the first laser pulse is launched, it generates a small-sized bubble within the droplet (t = 1 ms). At t = 2 ms, the introduction of another pulse leads to the formation of another bubble (highlighted within a dotted rectangle). Subsequent laser pulses generate new bubbles. These small bubbles subsequently coalesce to create larger bubbles with diameters of approximately 200 µm (Regime II). It is also possible that while a laser pulse primarily generates a bubble inside the droplet, the droplet's surface may rupture, as observed at t = 50 ms and 50.2 ms, initiating the breakup of droplet. Following the rupture of the droplet's surface, ligament stretching, and the eventual breakup occur, as evidenced by t = 50 ms (Regime II). The secondary droplets formed during the breakup process may coalesce, facilitated by the acoustic field employed for droplet levitation. The bubbles initially present in the parent droplet continue to coalesce, ultimately forming a larger bubble of approximately 600 µm in size (Regime III). Multiple pulses produce small bubbles which agglomerate during the laser pulse and bubble interaction to form large bubbles as seen in Fig. 3. The energy of the bubble is proportional to the cube of its maximum radius and can also be expressed in terms of laser energy ${E}_{l}$ as^54^

1 $$E_{B} = \frac{{4 \times \pi \times P_{a} }}{3} \times r^{3}_{max} = \gamma \times E_{l} ,$$

where P_a_ and $\gamma$ are pressure on the bubble by the liquid droplet and the fraction of the laser pulse energy converted to the bubble’s energy respectively. From Fig. 3, it is evident that bubbles generated by individual laser pulses coalesce and form a single larger bubble. The coalescence process depends on several physical parameters, including bubble size, the forces at play between two approaching bubbles, and the drainage time.

The approximated equivalent radius for two approaching bubbles can be defined as

2 $$R_{eq} = \frac{{2r_{1} r_{2} }}{{r_{1} + r_{2} }},$$

where ${r}_{1}$ and ${r}_{2}$ are the radii of two approaching bubbles. The size of these bubbles in the present study is in the range of 40–150 µm. As two bubbles approach each other, a thin layer of liquid forms in the contact region between them. This liquid film progressively grows in area until it reaches a critical thickness, at which point it ruptures. This rupture event results in the fusion of the two bubbles into a single, larger bubble, as depicted in Fig. 3 at t = 30 ms and t = 55 ms. The time from formation of contact area to complete fusion of bubble is often referred to as the coalescence time or film drainage time (${t}_{Ds}$), can be obtained using the equation formulated by Kirkpatrick and Lockett^55^

3 $$t_{Ds} = r_{f} \sqrt {\frac{{\rho_{l} \times R_{eq} }}{{16 \,\sigma_{l} }} \times ln\frac{{h_{0} }}{{h_{c} }}} ,$$

where ${r}_{f}$ represents the radius of the bubble contacting area, ${h}_{0}$ is the initial liquid film thickness, and ${h}_{c}$ is the critical film thickness at which the film ruptures. For numerical calculation, the properties of diesel fuel used are, density (${\rho }_{l}$) and surface tension (${\sigma }_{l}$) are 837 kg/m^3^ and 0.0258 N/m, respectively. The values for initial and critical film thicknesses typically fall within the range of 1–10 µm and 0.01 µm, respectively, as reported in the work by Oolman and Blanch^2^. For our analysis, we have adopted an initial film thickness (${h}_{0}$) of 6 µm and a critical film thickness (${h}_{c}$) of 0.01 µm.

**Figure 4:**
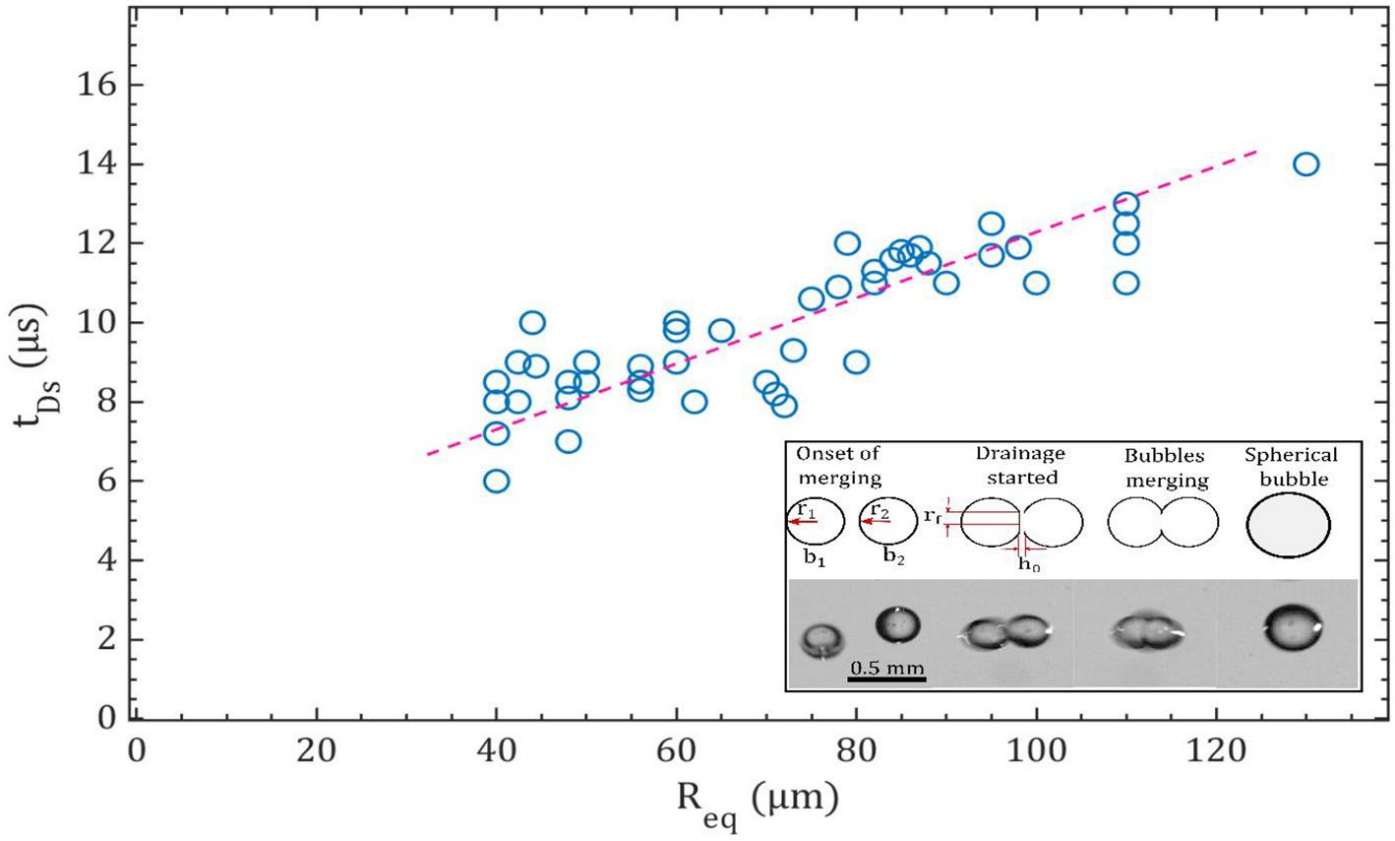
Variation of drainage time with equivalent bubble radius of diesel droplet. The inset provides a visual representation of the coalescence process and the parameters employed for the analysis.

The linear variation of drainage time with an equivalent bubble radius is observed in Fig. 4. This indicates that the time required for drainage during the coalescence of bubbles exhibit a linear increase with the equivalent radius of the bubbles. The coalescence process starts as two bubbles (b_1_ of radius ${r}_{1}$ and b_2_ of radius ${r}_{2}$) move close to each other. Drainage commences when these two bubbles reach a distance of ${h}_{0}$, marking the initial thickness of the interposed film between them, causing it to flatten. The film continues to drain until it reaches a critical thickness (${h}_{c}$< < ${h}_{0}$) and then ruptures at the radius of the contact area (R_f_). This process results in the formation of a single bubble and the corresponding drainage time ${(t}_{Ds})$ is an order of a few microseconds (~ 6 to 15 µs).

**Figure 5:**
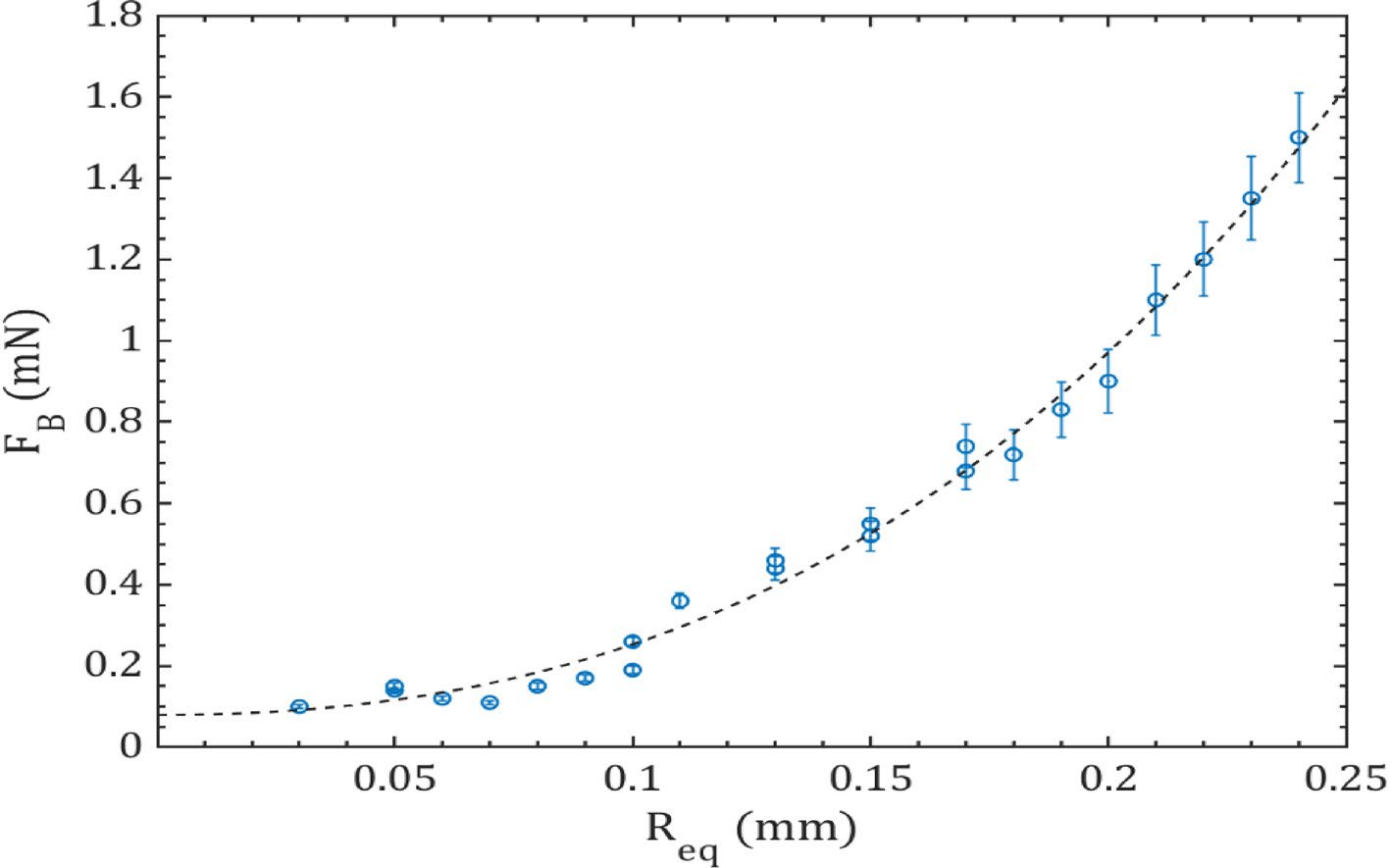
Variation of Bjerknes force with equivalent bubble radius for diesel fuel at E_l_ = 250 µJ.

Similar phenomena have been reported for acoustically generated bubbles^56^ and microbubbles generated by individual laser pulses for different liquids^11^. Several factors come into play when assessing the merging of bubbles, including the speed of their approach, the viscosity of the surrounding fluid, and the forces acting on microbubbles that lead to their coalescence. The interaction of coalescing or rebounding bubbles is considerably influenced by the generation of an acoustic field within the liquid caused by the laser pulse pressure. This acoustic field adds complexity to the coalescence process due to the Bjerknes forces exerted on the micro-bubbles. Bjerknes forces represent the translational forces acting on the bubbles within a sound wave and fall under the category of acoustic radiation force. External sound fields give rise to primary Bjerknes force, while secondary Bjerknes force emerges as an attractive or repulsive force between pairs of bubbles within the same sound field. These secondary forces result from the pressure field-generated oscillations of each bubble. An individual laser pulse creates the bubbles inside the droplet. The formed bubbles accelerate because of the strong pressure field generated by the laser pulse. As these bubbles approach each other, the secondary Bjerknes force is created between them. The final merger or coalescence of the bubbles depends on the Weber number and secondary Bjerknes force. The magnitude of the Bjerknes force depends on several factors, such as the size and shape of the bubbles, the frequency and intensity of the acoustic field, and the properties of the surrounding liquid. The coalescence of bubbles can occur due to both primary and secondary Bjerknes forces, depending on the size of the bubbles. In this study, the emphasis is on measuring the secondary Bjerknes force, which is responsible for bubble-to-bubble interactions. The secondary Bjerknes force can be expressed in terms of volume change in a single acoustic cycle, taking into account the radii and radial velocities of the two interacting bubbles^57–59^

4 $$F_{B} = \frac{4\pi \rho }{{r^{2}_{12} }} \left( {r_{1}^{2} u_{1} r_{2}^{2} u_{2} } \right),$$

where ${r}_{12}$ is the separation distance between the two micro-bubbles taken from the center of the bubbles, ${r}_{1}$_,_ and ${r}_{2}$ are the radii of the two approaching bubbles, and u_1_ and u_2_ are the radial velocities of the respective bubbles. Figure 5 shows that the Bjerknes force increases exponentially with the equivalent bubble radius. This means that larger bubbles experience stronger forces than smaller bubbles when subjected to the same acoustic field. The Bjerknes force between the bubbles in this case is between 0.05 to 2.50 mN. The behavior of the bubbles when subjected to the acoustic field can vary. Sometimes, the bubbles may bounce off each other, while other times they may coalesce. The bubble may collapse due to the impact of free boundary during bubble interaction. If it does not collapse, it slows down the motion of the bubbles. However, the major influence on the bubble interaction is caused by the laser-generated pressure field, which further creates a secondary Bjerknes force between the two bubbles. This coalescence or rebound of the approaching micro-bubbles can be predicted using a dimensionless Weber number (*We*), which is expressed in terms of ${\rho }_{l}$ (the density of the liquid), *U* (the velocity of the two approaching bubbles), σ (the surface tension) and R_eq_ (the equivalent radius of the bubbles), given by

5 $$We = \frac{{\rho_{l} UR_{eq} }}{\sigma }.$$

The values of *We* decide the coalescence or bounce back of the drops/bubbles. In this study, mostly $We<0.2$ hence the process of coalescence is dominant over the bounce back of the bubbles.

### Process of droplet stretching and coalescence

**Figure 6:**
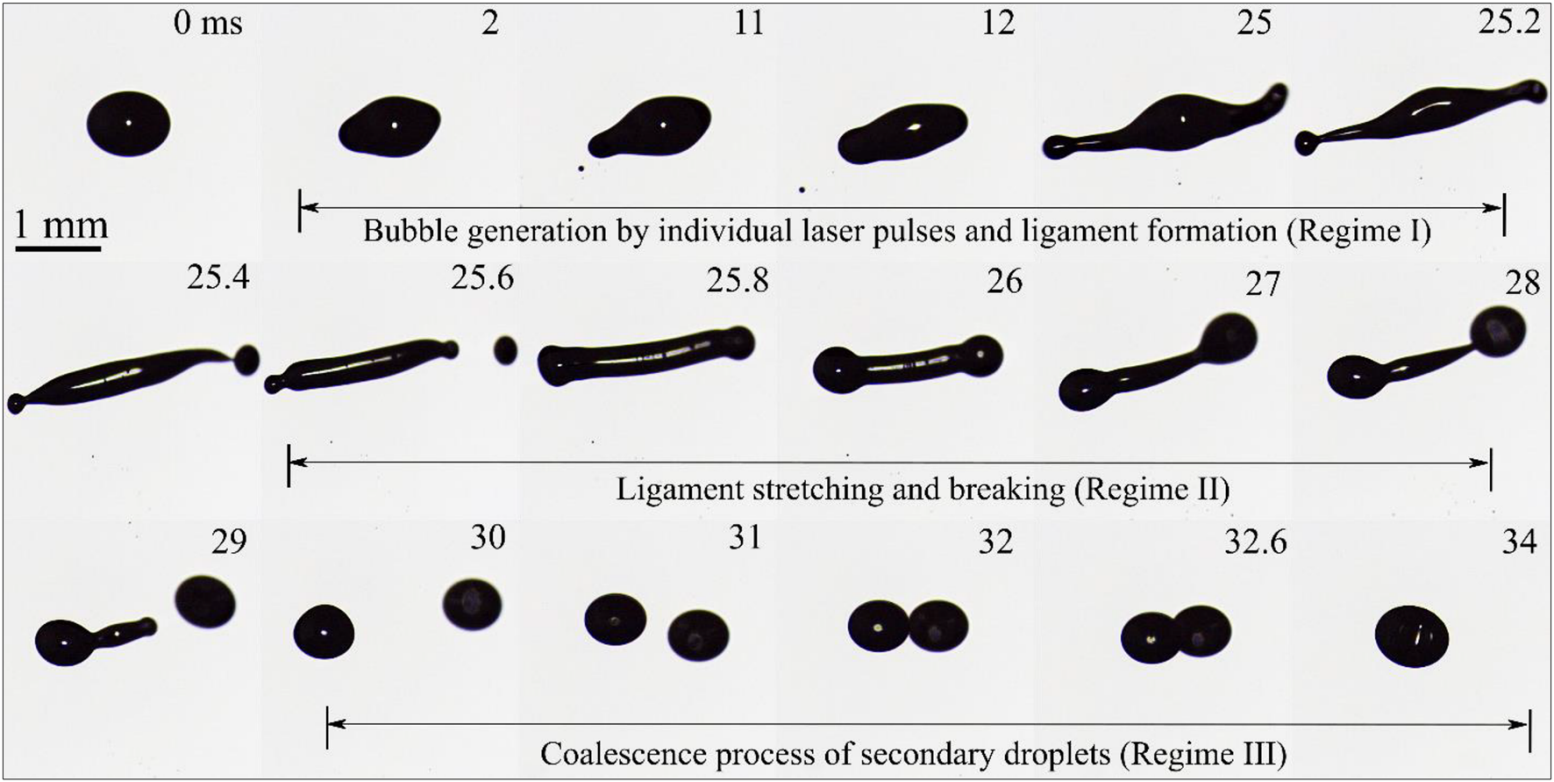
Ligament-mediated breakup in a diesel droplet through the interaction of multiple femtosecond laser pulses at E_l_ = 250 µJ and D_0_ = 0. 9 mm.

In this section, droplet stretching, and the coalescence process of secondary droplets are discussed. Like the process of bubble dynamics, this phenomenon can also be divided into three distinct regimes: I. Bubble formation II. Ligament stretching and its breakage and III. Secondary droplet coalescence. When the laser pulses interact with the droplet, multiple bubbles are generated by individual laser pulses. These bubbles grow and merge to form larger bubbles. An increase in the size of the bubble leads to the stretching of the droplet. The elongation/stretching of the droplet in the forward direction can be seen in Fig. 6 (shadow images of droplet) from the time interval t = 25 ms to 25.4 ms. The stretching and eventual rupture of the droplet results in the formation of a long thick ligament. This ligament undergoes the Rayleigh plateau instability and eventually breaks into several secondary droplets. Following the detachment of these secondary droplets from the ligament, they proceed to coalesce with each other, ultimately forming a single drop. The coalescence or rebound of approaching secondary drops is influenced by the external acoustic field, as illustrated in Fig. 6. The entire process of droplet coalescence can be divided into three consecutive stages (Regime III): (1) starting with their contact followed by the development of a thin bridge of fluid between them, (2) the subsequent contraction and breaking of the bridge, and (3) the growth of the connection neck between two droplets. In the first stage of coalescence (t = 31 ms), there exists a thin bridge between the two deformed droplets. Once this bridge breaks, a connection neck between two droplets is formed, and the process of coalescence is initiated (t = 32 ms), second stage. After the initiation of the connection between two droplets, the radius of the connection neck (t = 32.6 ms) increases. The growing speed of the neck results from a competition between the capillary forces driving the coalescence and opposing viscous forces. Finally, two droplets coalesce into one bigger droplet (t = 34 ms) in the third stage of the droplet coalescence process.

### Sheet breakup

In this section, droplet rupture followed by sheet formation and its breakup is discussed. At peak strength of 1050 µJ laser energy per pulse primarily results in the bubble generation inside the droplet and later fragmentation of the droplet through sheet breakup. Introduction of multiple laser pulses further generate multiple bubbles and leads to droplet ruptures. Afterward, the droplet will expand in a vertical direction and a liquid sheet is formed. During the sheet formation, multiple fragments come out from the edge of the sheet. It's important to note that the droplets of similar onset diameters of the different liquids may fragment at varying numbers of laser pulses. The sheet breakup is categorized into two primary types: a) Stable sheet breakup and b) Unstable sheet breakup.

#### Stable sheet breakup

**Figure 7:**
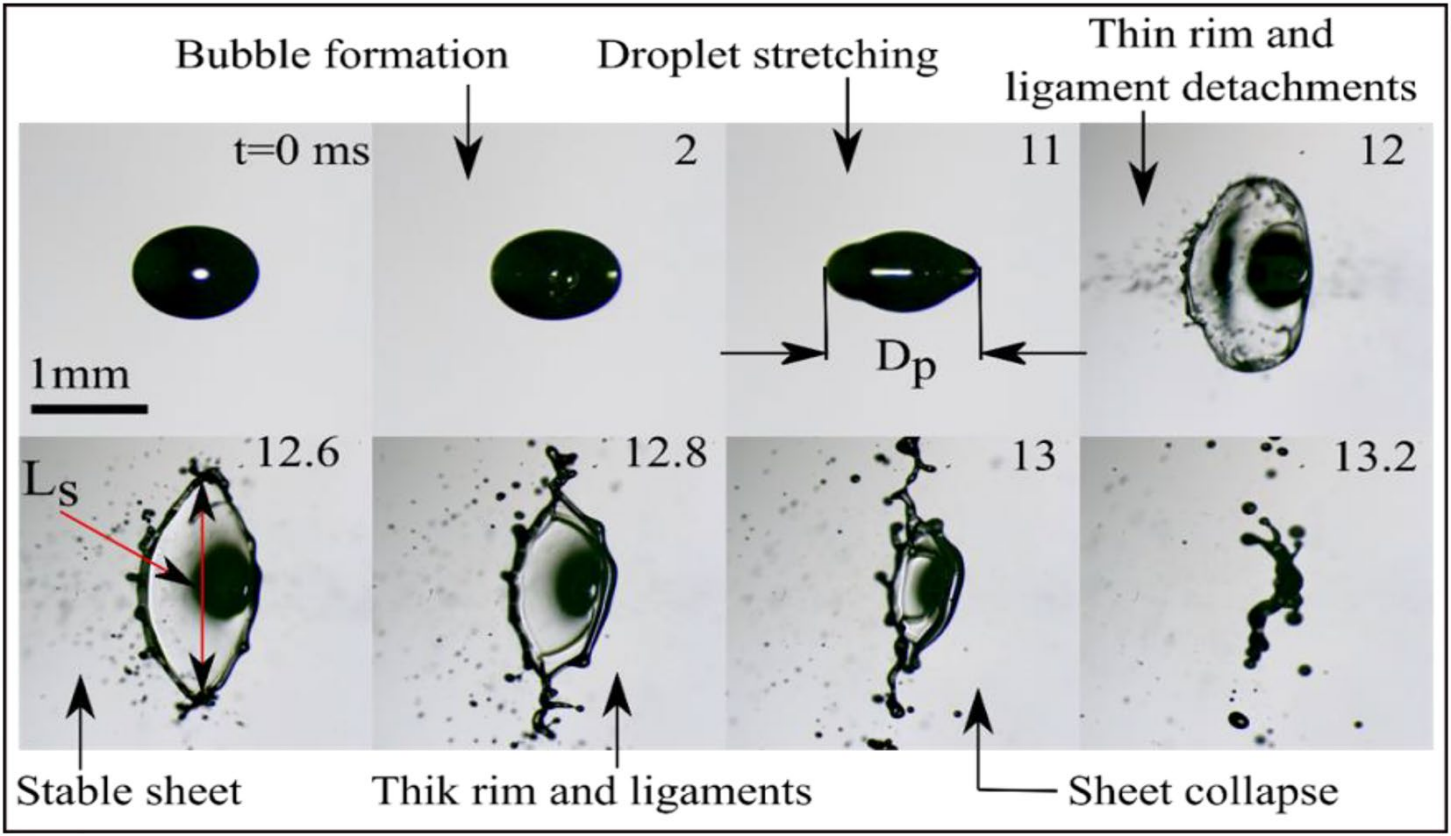
The process of stable sheet formation in a diesel droplet through the interaction of multiple femtosecond laser pulses at E_l_ = 1050 µJ and D_0_ = 1.1 mm.

The shadowgraph images in Fig. 7 show the sheet formation and its breakup of droplets of diesel subjected to a laser energy of 1050 µJ per pulse. The propulsion of the droplet depends on laser energy absorbed by the droplet which depends on local asymmetric boiling. This process leads to the formation of small vapor bubbles that break up and generate a thrust force on the droplet. It is important to note that the effects of laser radiation pressure and acoustic radiation pressure on the droplet during this process are insignificant. This means that the force generated by the vapor bubble formation and breakup is the main force responsible for propelling the droplet. The induced thrust force deforms and stretches the droplet (see at t ~ 11 ms) resulting in breaking the droplet with primary ligament detachment (t ~ 12 ms) and forming a thin rim. Further, the liquid sheet is expanded to form a stable thick sheet. During this process of expansion, the rapid acceleration of the sheet is observed which is in order of ɑ ~ 10^4^ m/s^2^. The very high acceleration of the sheet is prone to Rayleigh–Taylor instability. The growth of this instability is calculated by Villermaux and Clanet^60^, where ${\sigma }_{l}$ and ${\rho }_{l}$ are the liquid surface tension and density respectively.

6 $$\Delta t_{RT} = \left( {\frac{{\sigma_{l} }}{{\rho_{l} \times a^{3} }}} \right)^{1/4} ,$$

Theoretically, the calculated growth rate is ${\Delta t}_{RT} \sim 0.03 ms$ which is less than the experimental frame interval (0.2 ms). The initial (t ~ 12 ms) formed liquid sheet has a thin rim at the edge and a thick one at the center. Further, the gradual accumulation of the liquid at the edges of the sheet makes the sheet edge thicker and the rim diameter increases. This process leads to the forming of a more stable sheet having a thick rim and ligaments emerging from the edges (t ~ 12.6 ms). At the beginning of the process, small undulations form on the rim which may not be immediately discernible in the experimental observations. As the process continues, these undulations become more pronounced and develop into perturbations with a specific wavenumber, k_r_, from which ligaments begin to grow. The wavelength of the corrugations provides an experimental measure of the growth rate of the Rayleigh–Taylor instability on the rim. This instability mode, growing fast on the rim, is expressed by Klein et al*.*^4^ as

7 $$k_{r } \sim (a\rho_{l} /\sigma_{l} )^{1/2} .$$

The experimentally observed growth rate (6.12 ± 0.15 mm^−1^) is of approximately the same order as the theoretically calculated (18 mm^−1^) growth rate. Further, the formed ligaments undergo the Rayleigh-Plateau instability and eventually break up into smaller secondary droplets. The ligament breakup time is the time between the formation of the ligament and the pinching of the first droplet from the ligament. This experimental ligament breakup time is measured, and the value is compared to the capillary time scale of the ligament (${\tau }_{cL}$). This time is expressed in terms of surface tension, the density of the liquid, and the ligament diameter ${L}_{d}$, which is given by^51,61^:

8 $$\tau_{cL} = \sqrt {\frac{{\rho_{l} \times L_{d}^{3} }}{{\sigma_{l} }}} .$$

**Figure 8:**
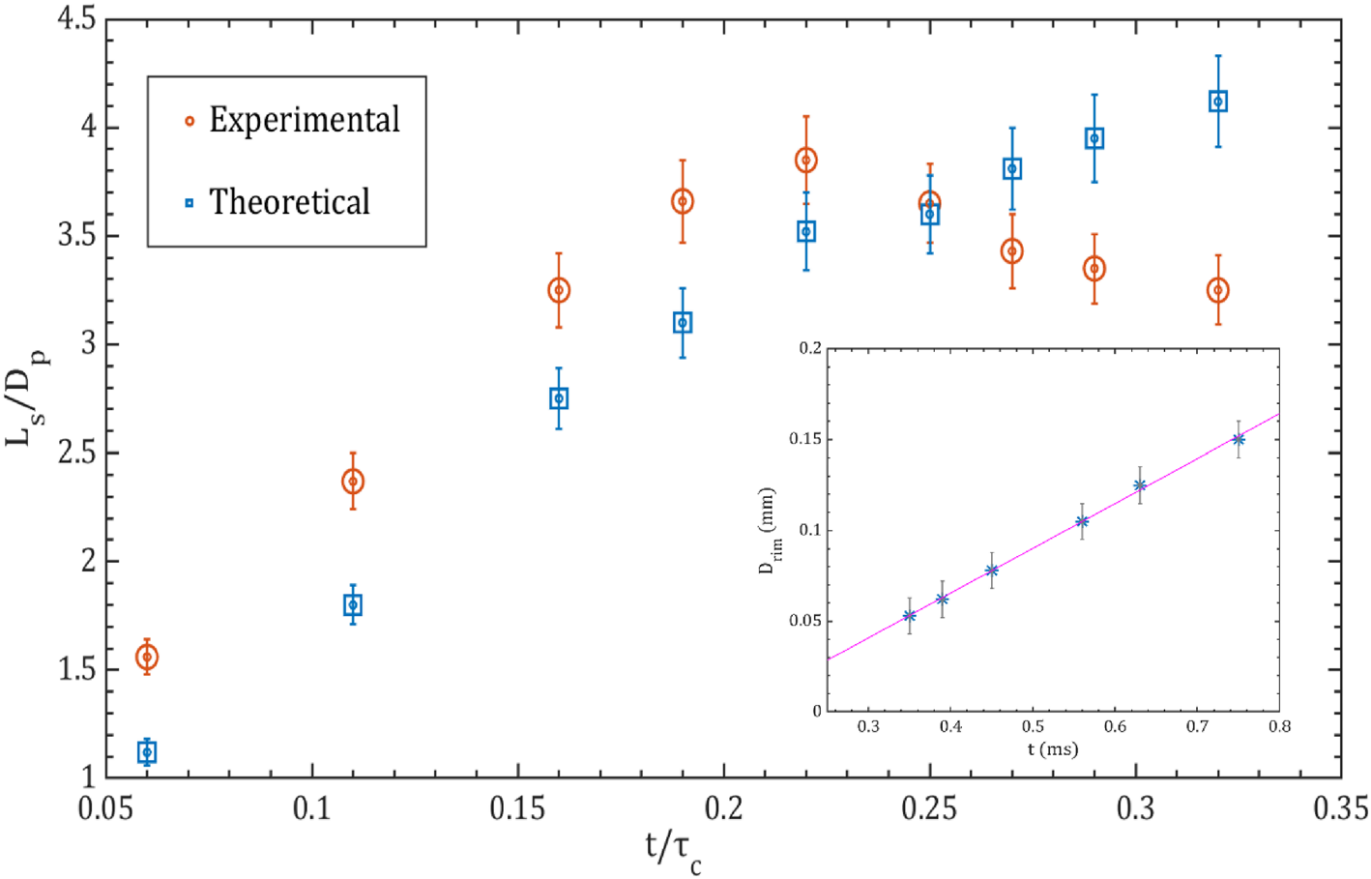
Experimental and theoretical comparison of temporal variation of the normalized length scale of the sheet. The inset represents the temporal variation of rim diameter for the stable sheet.

The theoretically measured ligament capillary time is 0.4 ms whereas the experimental breakup time observed is 0.6 ± 0.2 ms. Once the ligaments break, the sheet collapses under the influence of surface tension. The lifespan of the sheet is the time when the sheet collapses, hence the experimental collapse time is compared with the capillary time scale of the sheet. The capillary time is theoretically given by Avila and Ohl^48^.

9 $$\tau_{c} = \sqrt {\frac{{\rho_{l \times } D_{p}^{3} }}{{8 \sigma_{l} }}} ,$$

where D_p_ is the diameter of the liquid droplet at a pre-breakup instant. The theoretical capillary time calculated is 1.2 ms and the observed experimental sheet collapse time is 1.0 ms. Figure 8 describes the experimental and theoretical evolution of the stable sheet diameter over time, along with the increase in the rim diameter due to continuous liquid accumulation at the edge. The results obtained from the experiments for the evolution of the length scale of the sheet nearly match with the evolution of the sheet theoretically predicted. The inset in Fig. 8 depicts the linear relationship between the rim diameter and time. The theoretical model proposed by Klein et al.^5^ is used to explain the observed sheet evolution dynamics in the experimental study. A mathematical model can be used to describe the development of a stable liquid sheet over time is expressed as^3^

10 $$\frac{{L_{s} \left( t \right)}}{{D_{p} }} = 1 + \sqrt {3 We_{d} } \frac{t}{{\tau_{c} }} \left( {1 - \sqrt {3 } t/2\tau_{c} } \right)^{2} ,$$

where ${We}_{d}$ is given by

11 $$We_{d} = \frac{{E_{k,d} }}{{E_{k,cm} }} We.$$

    Here $\frac{{E}_{k,d}}{{E}_{k,cm}}$ is the ratio of deformation to propulsion kinetic energies which depends on the laser beam profile^5^. The Weber number (We) is a dimensionless parameter that describes the ratio of the kinetic energy of a droplet to the surface energy that resist the deformation of the droplet. In the case of sheet fragmentation caused by nucleated bubbles, the Weber number is modified because the presence of bubbles can change the surface tension of the liquid, which affects the energy required to deform the liquid sheet. However, under conditions of higher laser intensities, the nucleated bubble vanishes. In this scenario, the modified Weber number is defined as the displacement kinetic energy of the drop to its surface energy, which is given by

12 $$We \sim \frac{{\rho_{l} D_{p} U^{2} }}{{\sigma_{l} }},$$

Where U is the axial velocity of the sheet. The Weber number ($${We}^{*})$$ is sometimes rescaled to $${We}_{d}$$ to account for the energy required for deformation that is not available for propulsion. The ratio of kinetic energy to propulsion energy can be expressed in terms of the radial velocity inside the sheet $$({u}_{r} \sim r/{t}_{max} )$$, which is given by

13 $$\frac{{E_{k,d} }}{{E_{k,cm} }} \sim \frac{{\mathop \smallint \nolimits_{0}^{R} u_{r}^{2} r \,dr}}{{U^{2} \mathop \smallint \nolimits_{0}^{R} r \,dr}}.$$

Here, the time taken for the sheet to reach its maximum extension is represented by ${t}_{max}$ and the maximum radius of the stable sheet is denoted by ${R}_{{S}_{max}}$.

Then the ratio $\frac{{E}_{k,d}}{{E}_{k,cm}}$ can be written as

14 $$\frac{{E_{k,d} }}{{E_{k,cm} }} \sim \frac{{\left( {R_{{S_{max} }} } \right)^{2} }}{{2 U^{2} \left( {t_{max} } \right)^{2} }}.$$

**Figure 9:**
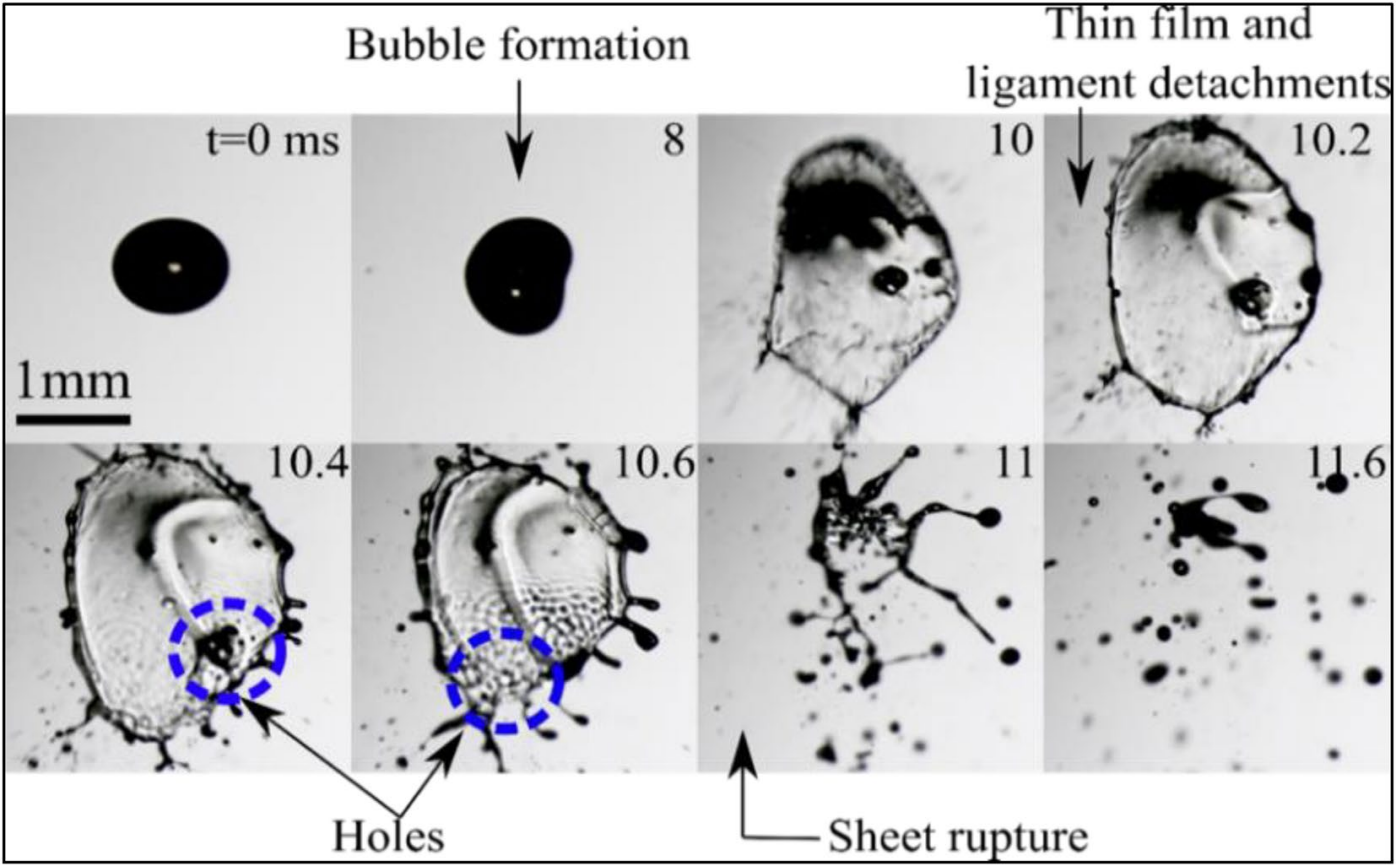
The process of unstable sheet breakup in a diesel droplet through the interaction of multiple femtosecond laser pulses at E_l_ = 1050 µJ and D_0_ = 0.95 mm.

The derived equations are based on energy conservation along the curved streamline, extending from the heart of the crushing droplet to the expanding sheet. However, when an expanding sheet reaches its maximum radius, the theoretically calculated time exceeds the experimentally observed time. After reaching its maximum expansion, the sheet undergoes deceleration and collapses due to surface tension forces. This deceleration leads to a reduction in the sheet's radius, consequently causing a decrease in the Weber number (W_ed_). As a result, there is a decrease in the normalized time for the experimental case. In essence, the discrepancy between theoretical and experimental times can be attributed to the dynamic behavior of the sheet, which involves both expansion and subsequent collapse, influenced by surface tension effects. The discrepancy in the normalized time scale arises from variations between the theoretical capillary time and the experimental sheet collapse time. Specifically, when *t*/*τ*_c_​ > 0.2 (Fig. 9), the theoretical capillary time scale surpasses the experimental sheet collapse time. Consequently, this leads to an augmentation in the normalized time scale for the experimental case, contributing to an increase in the length scale of the stable sheet. Conversely, the theoretical case exhibits the opposite trend, experiencing a decrease in the length scale of the stable sheet due to the theoretical capillary time scale being higher than the experimental sheet collapse time during this timeframe.

#### Unstable sheet breakup

The shadowgraph images displayed in Fig. 9 provide a chronological overview of the formation and subsequent breakup of unstable sheets generated from diesel droplets subjected to laser pulses with an energy of 1050 µJ. The early-time dynamics shows a phenomenon similar to that of the stable sheet breakup process. However, the late-time dynamics show the breaking process via unstable sheet formation. The breaking of the bubble induces the thrust force that deforms the droplet into a sheet (t_1_ ∼ 10 ms). This sheet evolves in an unstable manner, developing undulations and holes on its surface by t = 10.4 ms. The evolution of the hole causes localized thinning of the sheet and finally, it ruptures (t = 11 ms). The liquid ligaments are formed due to the merging of holes in the sheet and surface undulations, as visible at t = 10.6 ms in Fig. 9. These ligaments then become Rayleigh–Plateau unstable and form relatively large droplets as compared to the initial ejecta on the left-hand side of the droplet, as seen at t = 10 ms in Fig. 9. The evolution of the holes represented by growth rate/velocity of the holes. Hence, by measuring the velocity of the holes (V_H_), the local planner sheet thickness (H) can be obtained by Taylor-Culick law^48,62^

15 $${\text{H}} = 2 \sigma /\rho V_{H}^{2} .$$

#### Catastrophic breakup

**Figure 10:**
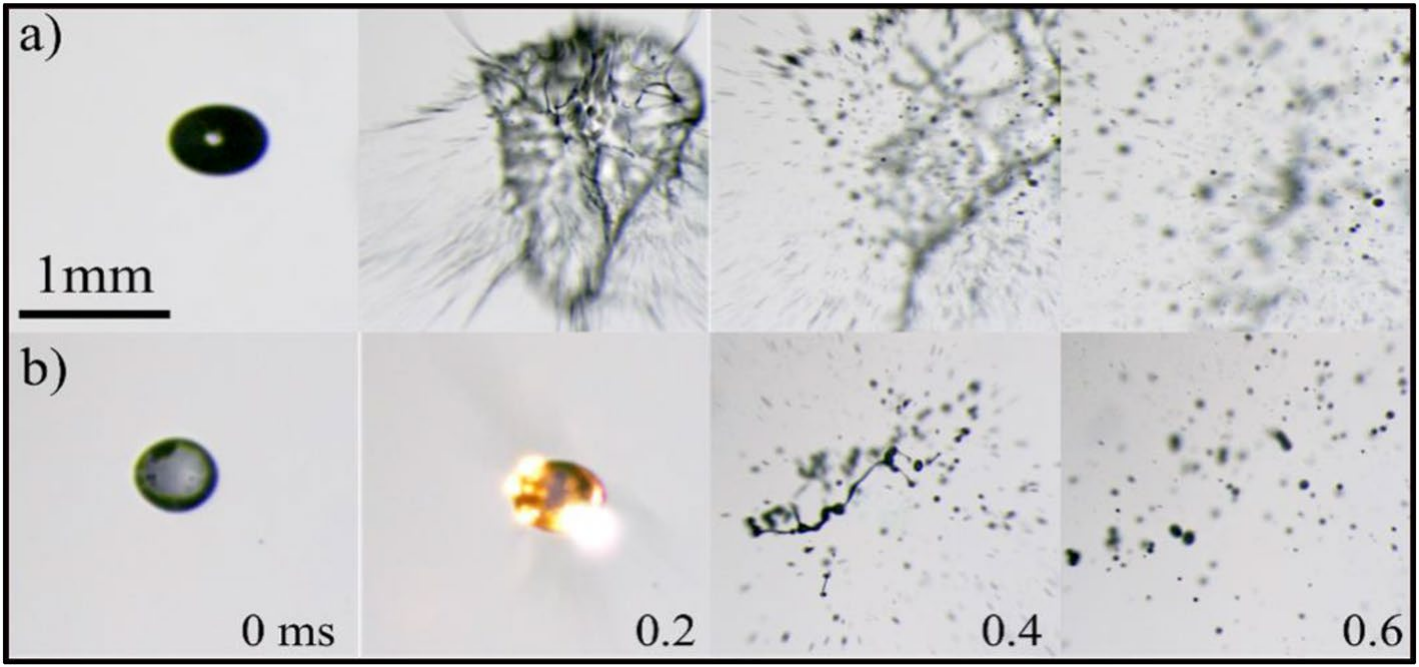
Catastrophic breakdown in a diesel droplet through the interaction of multiple femtosecond laser pulses at E_l_ = 1050 µJ and D_0_ = 0.55 mm.

Following the growth of a bubble within the droplet, the bursting of the bubble leads to catastrophic breakup of droplet generating secondary micro-droplets. Figure 10 shows the breakup process of the diesel droplet at 1050 µJ and the onset diameter of the droplet is D_0_ = 0.55 mm. The pressure force (F_p_) due to the breaking of the bubble can be expressed in terms of internal pressure (P_in_), ambient pressure (P_a_), and radius of the bubble (r_b_) is given as

16 $${\text{F}}_{{\text{P}}} \sim \left( {{\text{P}}_{{{\text{in}}}} - {\text{P}}_{{\text{a}}} } \right) \pi {\text{r}}_{{\text{b}}}^{2} .$$

Similarly, the surface tension force is expressed as

17 $${\text{F}}_{{\text{s}}} \sim \sigma_{l} \pi {\text{D}}_{{\text{p}}}$$

Weber’s number for droplet is of the order of 10^4^. The sizes and the velocities of the secondary droplets are in the range of ~ 10 to 80 µm and ~ 1 to 5 m/s respectively. The Pearson correlation^63^ is used to indicate the dependency of the secondary droplet size and its velocity. It is observed that the size of the secondary droplets increases, with corresponding decrease in velocity and vice versa. Hence it indicates that the secondary droplet size and the corresponding velocity are negatively correlated with a Pearson correlation coefficient of ~ -0.6. The empirical equation of the Pearson correlation coefficient^63^ is given as

18 $${\text{Correl}}\left( {{\text{D}}_{{\text{S}}} ,{\text{ V}}_{{\text{S}}} } \right) = \frac{{\sum \left( {{\text{D}}_{{\text{S}}} - {\overline{\text{D}}}_{{\text{S}}} } \right)\left( {{\text{V}}_{{\text{S}}} - {\overline{\text{V}}}_{{\text{S}}} } \right)}}{{\sqrt {\sum ({\text{D}}_{{\text{S}}} - {\overline{\text{D}}}_{{\text{S}}} )^{2} \sum ({\text{V}}_{{\text{S}}} - {\overline{\text{V}}}_{{\text{S}}} )^{2} } }},$$

where ${\overline{{\text{D}}} }_{{\text{S}}}$ and ${\overline{{\text{V}}} }_{{\text{S}}}$ are the average diameter and average velocity of the secondary droplets, respectively. Note that similar breakup patterns are observed for larger droplets when the laser energy exceeds 1050 μJ, which will be considered in our future investigations.

**Figure 11:**
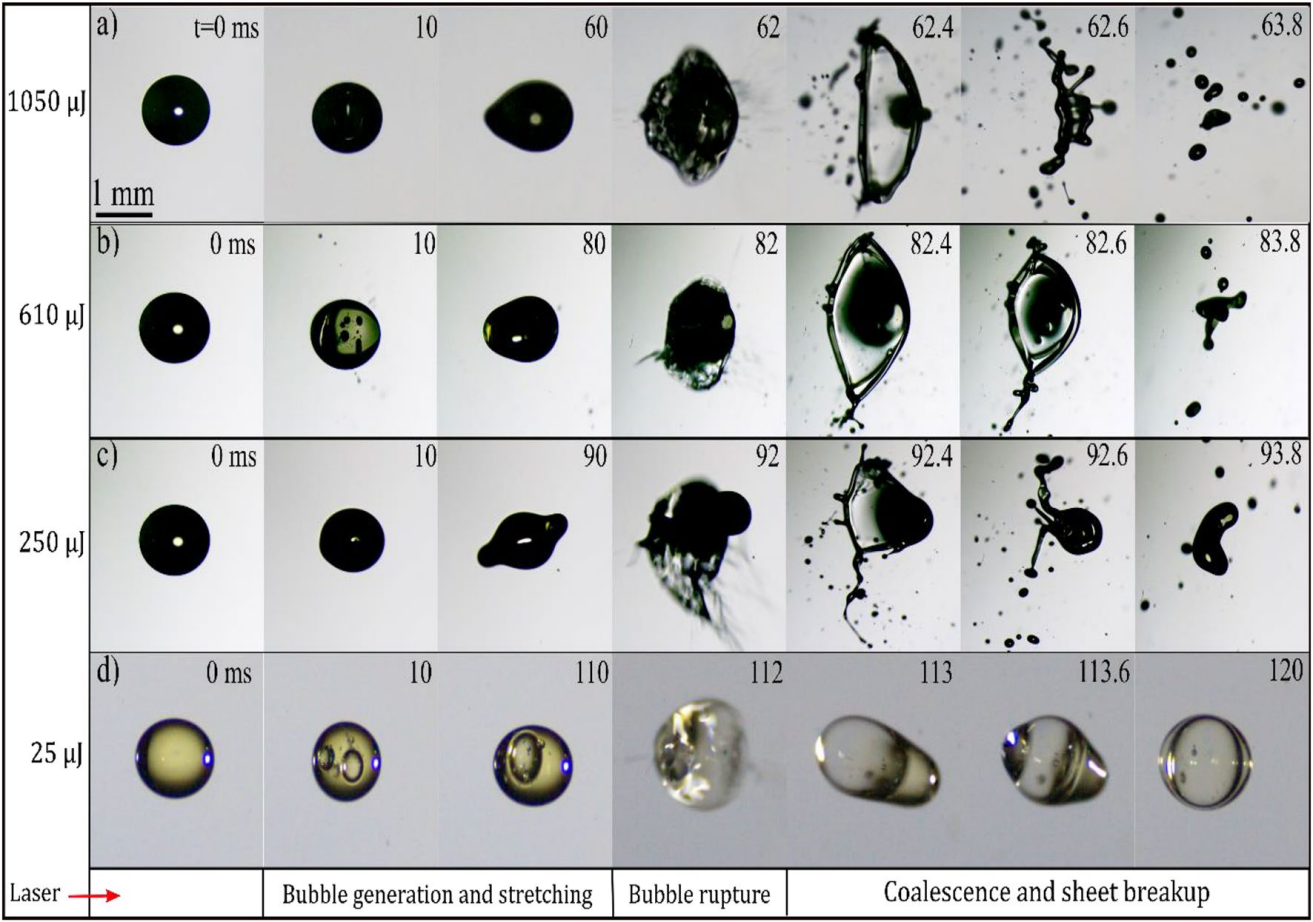
Time sequence images of RME droplet showing bubble dynamics and breakup through the interaction of multiple femtosecond laser pulses for different laser energy (E_l_) and D_0_ = 1.0 mm.

Figure 11 presents the evolution of an RME (Rapeseed Methyl Ester) droplet under varying laser energies. Similar to the phenomenon observed in diesel droplets, the process involves bubble dynamics followed by sheet breakup in the case of RME. However, the key difference is longer time required for breakup compared to that of the diesel. At lower energy levels (Fig. 11d), the process includes bubble generation, droplet stretching, and coalescence. As we increase the energy, dynamics change to sheet formation and breakup (Fig. 11a–c). When a bubble expands, it causes the rupture of a thin liquid film. This process can lead to the formation of submicron-sized droplets. For smaller droplets, the film separating the expanding bubble from the atmosphere may rupture earlier, resulting in a more violent acceleration of the droplet body. This can lead to the formation of submicron-sized droplets and a coarser fragmenting front part of the droplet. This process is likely caused by Rayleigh–Taylor's instability. On the other hand, larger droplets or moderate energies may later result in the film rupturing. In this scenario, the pressure difference between the expanding bubble and the atmosphere results in a less violent acceleration of the droplet body, causing it to deform into a sheet. The behavior of the sheet depends on its size and surface tension. Smaller sheets are dominated by surface tension and collapse back into a droplet, while larger and thinner sheets may rupture, leading to Rayleigh-Plateau unstable liquid films. For even larger droplets or lower energies, the droplet may remain intact with only minimal loss of mass.

#### Secondary droplet size distribution

**Figure 12:**
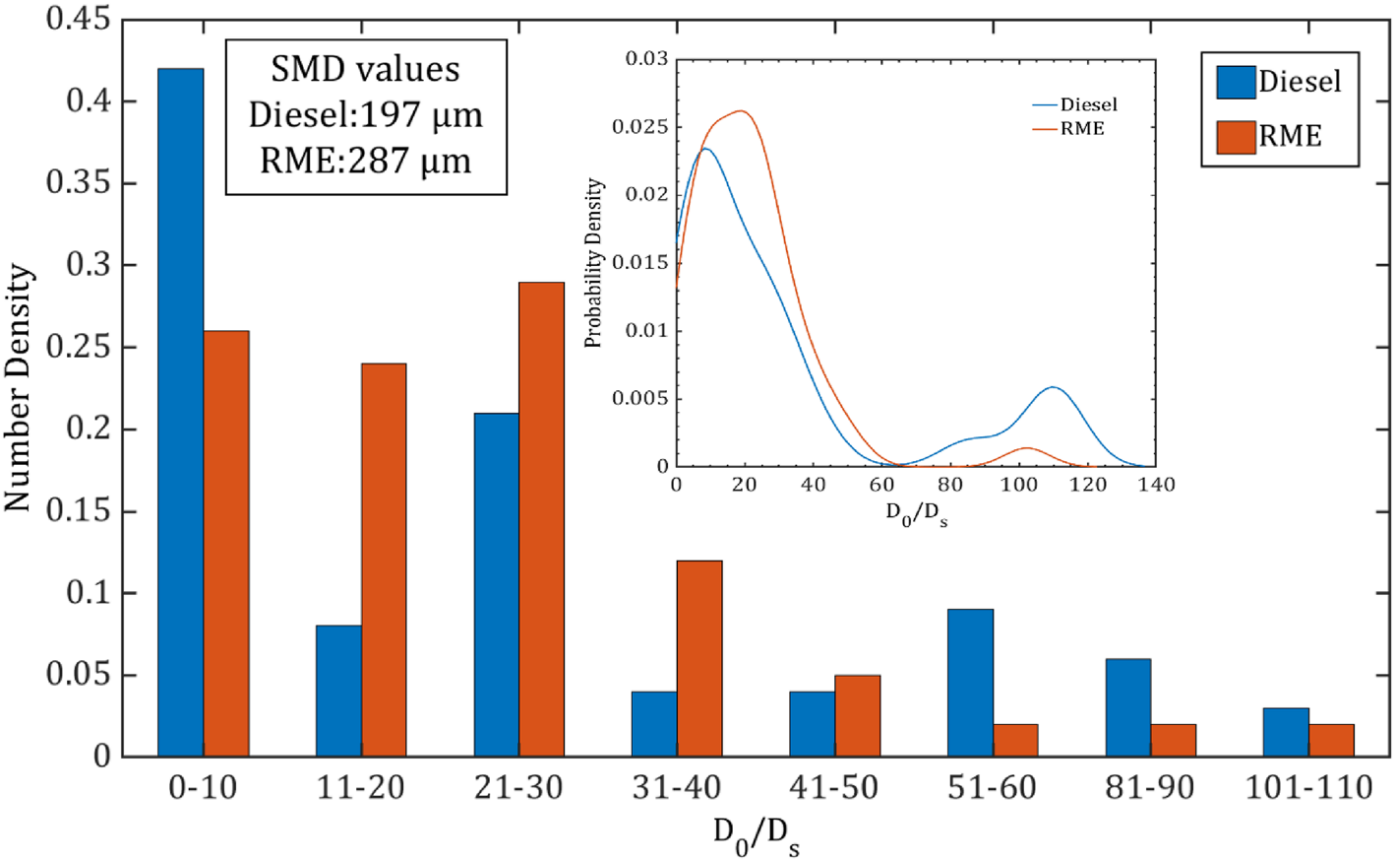
Normalized secondary droplet size distribution of the diesel and RME corresponding to a laser pulse energy of 1050 µJ, and onset droplet diameter of D_0_ = 1.0 mm. Inset inside the Fig. 12 represents the probability density function plotted against normalized secondary drop size for diesel and RME droplets. Where D_*s*_ and D_0_ are the secondary and onset droplet diameters respectively.

Figure 12 illustrates the normalized secondary droplet size distribution for diesel and RME droplets at an energy level of 1050 µJ. There's a discernible difference in the trend of droplet size distribution between diesel and RME. The secondary droplets, primarily generated by ejected fragments, tend to be smaller than the droplets formed through sheet breakup. The Sauter Mean Diameter (SMD) values for diesel and RME represent the average size of the droplets generated after the breakup process.

**Figure 13:**
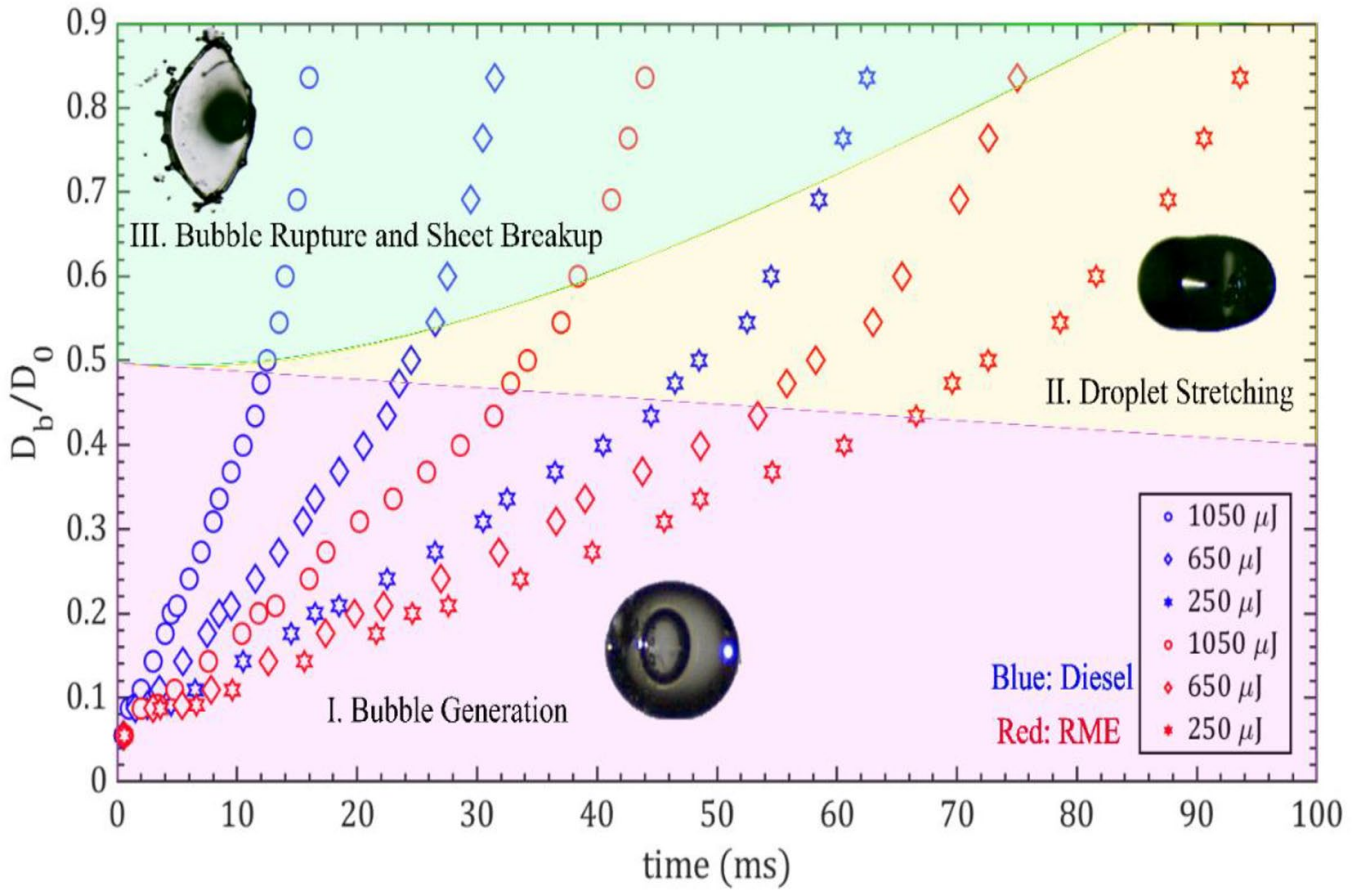
Regime map of temporal variation of a normalized bubble size distribution at different laser pulse energies for the diesel and RME droplet.

The bubble generation and droplet breakup process observed above are summarized in Fig. 13. The three different regimes observed in the process of bubble formation and breakup from the diesel and RME droplets experiments are shown. The first regime i.e. bubble generation is observed at a small D_b_/D_o_ ratio, which indicates the generation of multiple bubbles for both fuels. The time taken for bubble generation of both fuels at lower laser energy levels is higher. As the ratio D_b_/D_o_ increases, the regime changes to droplet stretching (regime II: yellow shade) and subsequently undergoes bubble rupture and sheet breakup (regime III: represented in pink color). In all the cases, the time required for RME is generally longer compared to that of diesel fuel. Regime III is observed at a higher D_b_/D_o_ ratio and at a moderate ratio i.e. only droplet stretching occurs. Note here that, the time required for the breakup of both the liquid droplets decreases with increasing laser energy. The graphs show the different regimes of bubble formation and time as a function of laser energy and effect of liquid properties. Increasing the laser energy reduces the time for bubble formation and quickly shifts to droplet rupture and sheet breakup. The physical properties of RME (absorption coefficient, surface tension and viscosity) delays the process of bubble formation, stretching and breakup. The changes in the regimes from bubble dynamics to sheet breakup are presented in Table 2. Initially, the bubble dynamics is observed at all laser energy and lower ratio D_b_/D_0_ for both diesel and RME fuels. For D_b_/D_0_ > 0.5, sheet breakup is observed at 750 µJ and 1050 µJ laser energy for diesel, while in the case of RME, it is observed only at a higher laser energy of 1050 µJ. Droplet stretching is observed from D_b_/D_0_ > 0.4 at all energy for RME and diesel, specifically at 250–750 µJ laser energy (Table 2).

**Table 2:** Detailed description of regime map presented in the Fig. 13.

Regimes	D_b_/D_0_	Laser energy	Liquids
I. Bubble generation	< 0.5	All	Both
II. Droplet stretching	> 0.4	250 µJ	Both
		750 µJ	Both
		1050 µJ	RME
III. Bubble rupture and sheet breakup	> 0.5	750 µJ	Diesel
		1050 µJ	Both

**Figure 14:**
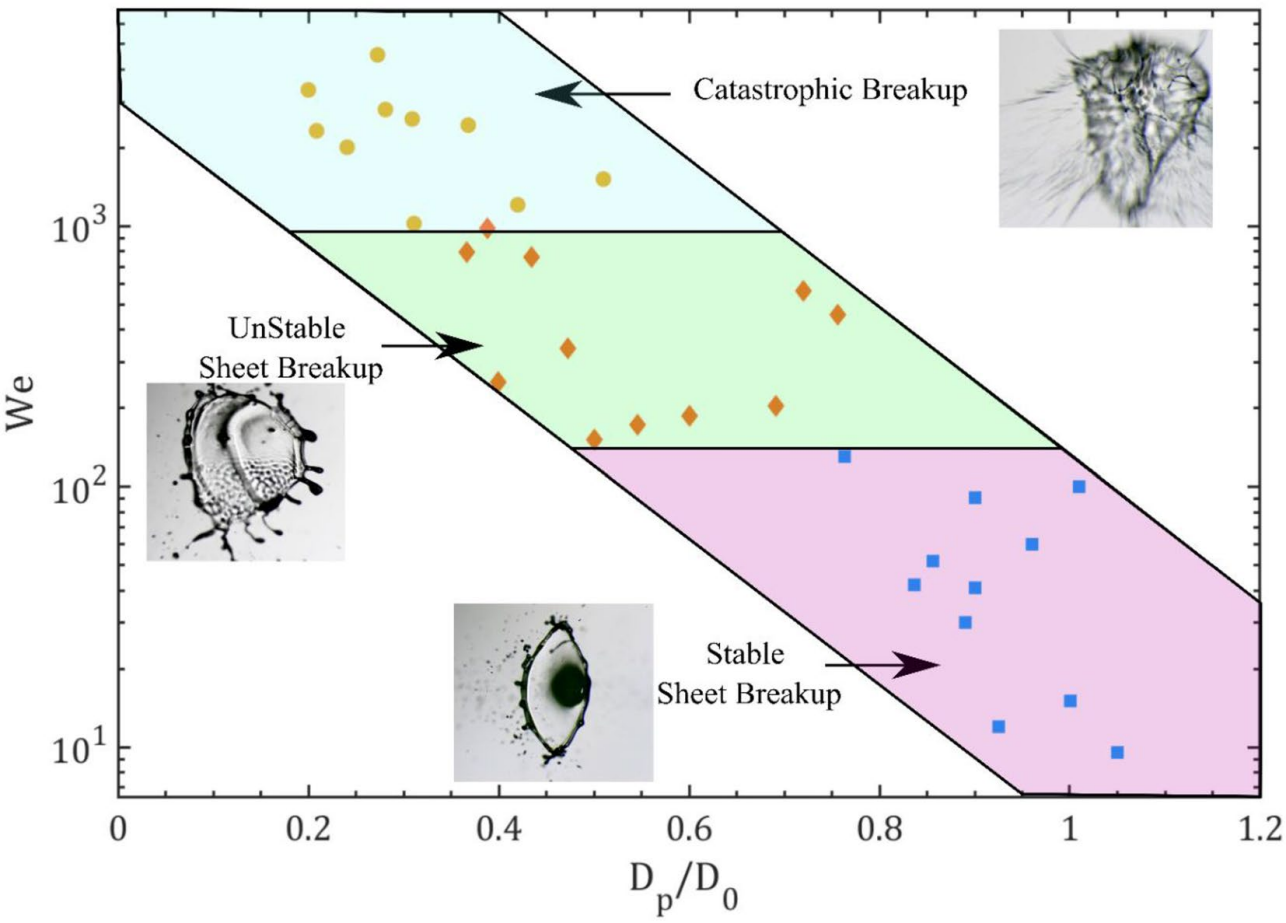
The detailed mapping of breakup features observed in the current study.

The detailed mapping of the breakup features is illustrated in Fig. 14. In this work, we observed the breakup feature changes from low strength stable sheet to high strength catastrophic breakup depending on the laser energy and diameter at a pre-breakup instant. The mapping of the breakup mode is decided by the ratio D_p_/D_0_ and Weber number, We as shown in Fig. 14. The stable sheet breakup is observed at a higher ratio D_p_/D_0_ and lower We whereas the lower ratio D_p_/D_0_ and higher We corresponds to unstable sheet breakup. When the bubble breaks, the impulse pressure is applied on the drop surface. Because of this, the fluid motion develops inside the droplet. The propulsion of the liquid sheet represented by axial velocity U follows the global mass conservation given as

19 $$P_{i} \tau_{i} D_{p}^{2} \sim \rho_{l} D_{p}^{3} U,$$

where

20 $$U \sim \frac{{{\text{P}}_{{\text{i}}} {\uptau }_{{\text{i}}} }}{{{\uprho }_{l} D_{p} }} .$$

Here P_i_ denotes the impulse pressure on the droplet after bubble breakup, U is the axial velocity of the sheet, and ${\uptau }_{{\text{i}}}$ represents the time scale on which impulse acts. In this study, for same-size bubble breakup the impulse pressure P_i_ and time scale ${\uptau }_{{\text{i}}}$ remain constant. Hence the axial velocity of the sheet is approximated to be inversely proportional to the pre-breakup diameter and expressed as

21 $$U \sim {\raise0.7ex\hbox{$1$} \!\mathord{\left/ {\vphantom {1 {D_{p} }}}\right.\kern-0pt} \!\lower0.7ex\hbox{${D_{p} }$}}.$$

Therefore, from Eqs. (12) and (21), the Weber number is expressed as

22 $$We \sim {\raise0.7ex\hbox{$1$} \!\mathord{\left/ {\vphantom {1 {D_{p} }}}\right.\kern-0pt} \!\lower0.7ex\hbox{${D_{p} }$}}.$$

Hence the dependency of the We are given by the Eq. (22) which shows the stable sheet corresponds to the higher ratio ‘D_p_/D_0_’ and lower ‘We’, whereas the unstable sheet breakup corresponds to the lower ratio ‘D_p_/D_0_’ and higher ‘We’. More violent fragmentation of droplets is observed at very high We and lower ratio which is represented as catastrophic breakup. Hence for constant onset diameter of the droplet, the weber number depends on the prebreakup diameter of the droplet. Therefore the D_p_ is deciding parameter for behavior of the breakup. The breakup behavior changes with liquid properties and laser pulse energy. In summary, The transition of the weber number with droplet ration for different liquids describe the breakup behavior and its strength.

## Conclusions

The present study delineates the distinct characteristics of femtosecond laser-induced bubble dynamics and the accompanying breakdown of diesel and RME droplets. We reveal their spatio-temporal evolution as a function of laser pulse energies (∼25 to 1050 μJ) for liquid droplets. Key findings and observations include:

1. *Energy dependence*: Both bubble diameter and breakup exhibit a logarithmic relationship with laser energy, irrespective of the liquid. At lower laser energy levels, smaller-sized bubbles are generated with each pulse. However, with increasing laser energy, the film that separates the expanding bubble from the atmosphere may rupture earlier. This results in a more violent acceleration of the droplet body, leading to the formation of submicron-sized droplets. This process is accompanied by the release of a shock wave into the surrounding air and the fragmentation of the original droplet, likely caused by Rayleigh-Taylor instability.
2. *Micro-bubble formation*: The ligament generation and size distribution of micro-bubbles are influenced by adjusting the laser pulse energy and the number of laser pulses. Consecutive pulses lead to strong interactions and coalescence of pulsating bubbles through the Bjerknes force, typically in the range of 0.2 to 1.5 mN, due to the generation of a laser-induced acoustic field.
3. *Temporal dynamics*: Regardless of the laser energy used (E_l_), the early stages of the process involve bubble generation and the stretching of the droplet. In contrast, the later stages exhibit the breaking of the droplet through sheet formation and catastrophic breakup.
4. *Sheet formation and behavior*: In the case of larger droplets or when using moderate laser energies, the film may rupture later, resulting in a less violent acceleration of the droplet body. This, in turn, causes the droplet to deform into a sheet. The behavior of the sheet depends on the liquid's surface tension and the size of the sheet. Smaller sheets are primarily influenced by surface tension, causing them to collapse back into a droplet. Conversely, larger and thinner sheets are more prone to rupture, leading to the formation of Rayleigh-Plateau unstable liquid films.

In summary, this study sheds light on the intricate dynamics of laser-induced bubbles and their impact on droplet breakup, providing valuable insights into the behavior of different fuels under varying laser energy conditions. The phenomenon of bubble dynamics is nicely explained from the generation of bubbles to its evolution. The use of a femtosecond laser to generate microbubbles allows for exact control and prediction of the size, position, and polydispersity of the generated bubbles, making them useful in applications such as microemulsions^38,49^, laser-induce fragmentation of polymers^1,64^, and laser induced cavitation in e-fuels^6,48,51^. Furthemore, the findings could help in understanding the incubation effect and optimizing laser parameters for medical laser and nano/micro-object manipulation applications. Finally, the fragmentation study of diesel and biofuels can help to optimize experimental parameters for fuel to improve combustion efficiency.

## Data Availability

The datasets used and/or analysed during the current study are available from the corresponding author on reasonable request.
